# Supplementary material for: Fine-tuning molecular mechanics force fields to experimental free energy measurements
Source: bioRxiv. 2025 Jan 8:2025.01.06.631610. Preprint. [Version 1] doi: 10.1101/2025.01.06.631610 (PMC11741335; doi:10.1101/2025.01.06.631610)
Supplement: 1 [file NIHPP2025.01.06.631610V1-supplement-1.pdf]

bioRxiv preprint doi: https://doi.org/10.1101/2025.01.06.631610; this version posted January 8, 2025. The copyright holder for this preprint (which was not certified by peer review) is the author/funder, who has granted bioRxiv a license to display the preprint in perpetuity. It is made available under aCC-BY 4.0 International license.

# A Supporting Information

## A.1 Code Availability

The Python, C++, and CUDA code used to perform hydration free energy experiments and fine-tuning in this paper is distributed open source under Apache License, Version 2.0 at https://github.com/dominicrufa/timemachine as a fork from https://github.com/proteneer/timemachine. Core dependencies include PyTorch 2.0.0 [77], Deep Graph Library 0.6.0 [109], the Open Force Field Toolkit 0.11.2 [67], JAX 0.4.30 [12], and timemachine [117].

The submission scripts and notebooks associated with data generation, analysis, and visualization are found at https://github.com/dominicrufa/timemachine/tree/master/data

bioRxiv preprint doi: https://doi.org/10.1101/2025.01.06.631610; this version posted January 8, 2025. The copyright holder for this preprint (which was not certified by peer review) is the author/funder, who has granted bioRxiv a license to display the preprint in perpetuity. It is made available under aCC-BY 4.0 International license.

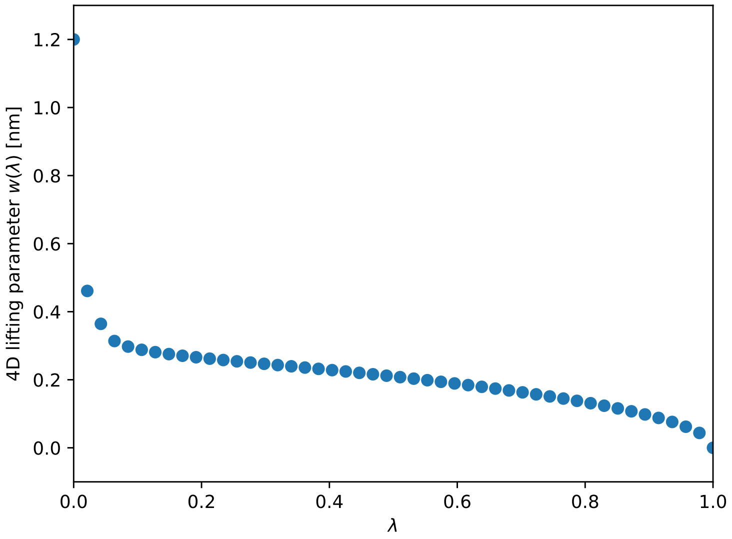


**Figure S 6. 4D coupling protocol $w(\lambda)$ as a function of $\lambda$.** $\lambda = 0$ corresponds to the decoupled state wherein the small molecule is non-interacting with TIP3P solvent as the 4-dimensional decoupling dimension is equal to $r_{\text{cut}}$. The 4-dimensional distance shrinks as $\lambda$ progresses to 1, where no interactions are lifted.

## A.2 Detailed Methods

### MM force field implementation

Bond, angle, and proper torsion parameters for FreeSolv small molecules were generated from the espaloma-0.3.2 foundation model and passed to the appropriate timemachine [117] energy/force evaluation functions. Openff-2.1.0 ("Sage") [27] improper torsions were used to replace espaloma-0.3.2 impropers due to conflicting conventions in atom ordering.

### FreeSolv hydration free energy calculation protocol

To compute hydration free energies for the FreeSolv dataset [67], we used a modified version of the protocol described in [70].

Neutral molecules were solvated with TIP3P water [50] in cubical boxes of width 4nm. Hydrogen Mass Repartitioning [44] was implemented between all heavy atoms and hydrogens by subtracting 2 amus from the former and adding to the latter.

A palindromic BAOAB Langevin integrator [57–59] was used with a friction coefficient of 1 $\text{ps}^{-1}$ and a timestep of 2.5 fs. A Monte Carlo Barostat at a temperature of 300 K and a pressure of 1.013 bar was used which alternated with MD steps.

Lambda independent dynamics were used to evaluate free energies between $\lambda = 0$ and 1 using Bennett Acceptance Ratio (BAR) differences between adjacent windows. Each window ran 5000 frames (at 400 timesteps per frame saving frequency) preceded by 10,000 equilibration timesteps with 48 $\lambda$ windows. The lifting term, $w(\lambda)$, in Eq. 4 is given by **Figure 6**, which is a pre-calibrated coupling schedule

## A.3 Fine-Tuning by Zwanzig reweighting

Algorithm 1 describes the ESS monitoring procedure of BFGS optimization associated with the grid of ESS-regularized, Zwanzig-reweighted fine-tuning refitting experiments in **Figure 7**. gtol is the default value set by `scipy.optimize.minimize` with `method = 'BFGS'`.

bioRxiv preprint doi: https://doi.org/10.1101/2025.01.06.631610; this version posted January 8, 2025. The copyright holder for this preprint (which was not certified by peer review) is the author/funder, who has granted bioRxiv a license to display the preprint in perpetuity. It is made available under aCC-BY 4.0 International license.

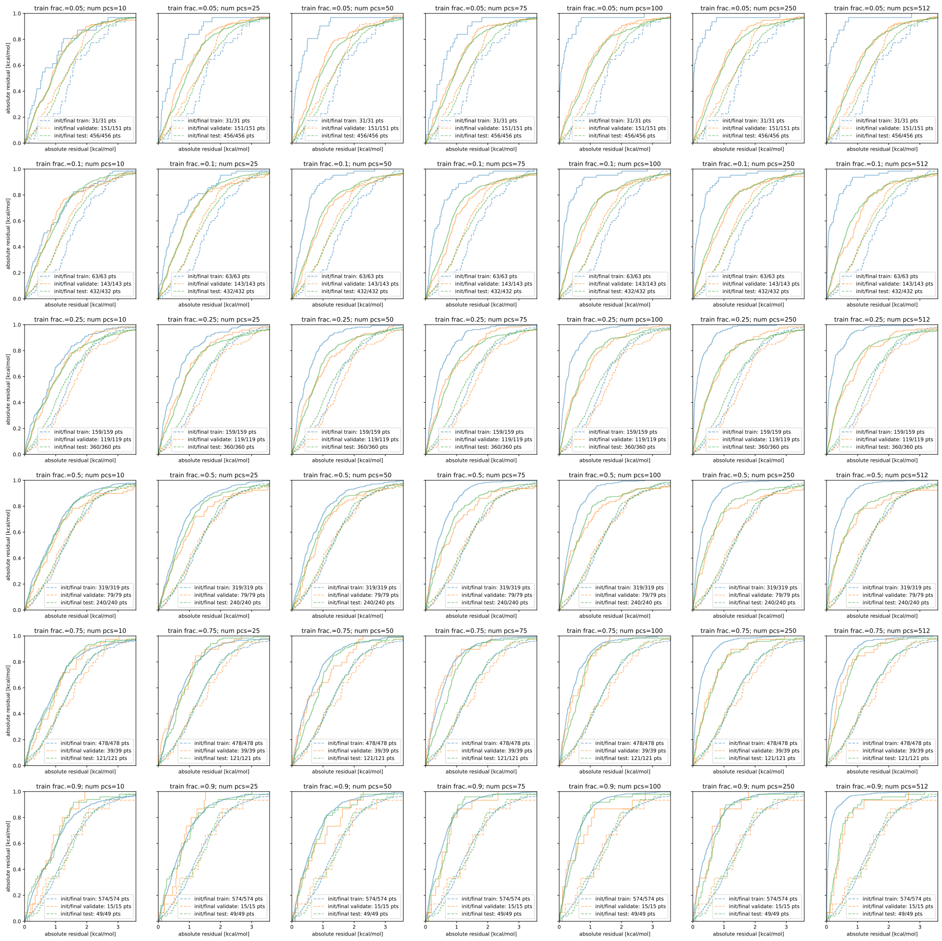

**Figure S 7. Full empirical CDFs of each ESS-regularized, Zwanzig-reweighted fine-tuning experiment** The aggregated RMSD and Cramer-von Mises statistics for each fine-tuning experiment are computed from the CDFs shown above. Training fractions and the number of PCs are depicted as graph titles above each plot. The number of data points pre- and post- optimization for each data partition that satisfy the ESS criterion are shown. Importantly, ESS regularization retains **all** data points in every partition for all experiments. The rightmost column (i.e., 512 principal components) did not include the change-of-base procedure from Eq. 6 since the dimension of each embedding is already of dimension 512 (i.e., $r_{max}$)

bioRxiv preprint doi: https://doi.org/10.1101/2025.01.06.631610; this version posted January 8, 2025. The copyright holder for this preprint (which was not certified by peer review) is the author/funder, who has granted bioRxiv a license to display the preprint in perpetuity. It is made available under aCC-BY 4.0 International license.

**Algorithm 1** BFGS optimization with effective sample size (ESS) monitoring

**Input:** Initial parameters $\mathbf{\Theta}$, ESS threshold $\text{ESS}_0 = 500$, gradient tolerance gtol
**for** `iteration` in range(1, 1001) **do** ▷ Maximum 1000 steps
  $\mathbf{\Theta}' \leftarrow \text{BFGS}(\text{Loss}(\mathbf{\Theta}))$ ▷ Compute updated parameters using BFGS optimization
  $\text{ESSs}_{\text{train}} \leftarrow$ compute ESSs of training set at $\mathbf{\Theta}'$
  $\text{ESSs}_{\text{test}} \leftarrow$ compute ESSs of test set at $\mathbf{\Theta}'$
  $\text{ESSs}_{\text{val}} \leftarrow$ compute ESSs of validation set at $\mathbf{\Theta}'$
  **if** `any`$(\text{ESSs}_{\text{train}}, \text{ESSs}_{\text{test}}, \text{ESSs}_{\text{val}}) < \text{ESS}_0$ **then**
    **return** $\mathbf{\Theta}$ ▷ Stop and return current parameters (ESS too low)
  **else if** $\|\nabla \text{Loss}(\mathbf{\Theta}')\| < \text{gtol}$ **then**
    **return** $\mathbf{\Theta}'$ ▷ Stop and return optimized parameters (convergence reached)
  **end if**
  $\mathbf{\Theta} \leftarrow \mathbf{\Theta}'$ ▷ Update parameters
**end for**
